# Human Endogenous Retroviral Envelope Protein Syncytin-1 and Inflammatory Abnormalities in Neuropsychological Diseases

**DOI:** 10.3389/fpsyt.2018.00422

**Published:** 2018-09-07

**Authors:** Xiuling Wang, Jin Huang, Fan Zhu

**Affiliations:** ^1^Department of Medical Microbiology, School of Medicine Wuhan University, Wuhan, China; ^2^Department of Medical Laboratory, The Central Hospital of Wuhan Huazhong University of Science and Technology, Wuhan, China; ^3^Key Laboratory for Molecular Diagnosis of Hubei Province, The Central Hospital of Wuhan Huazhong University of Science and Technology, Wuhan, China; ^4^Hubei Province Key Laboratory of Allergy and Immunology Wuhan University, Wuhan, China

**Keywords:** HERV, syncytin-1, inflammation, schizophrenia-associated genes, neuropsychological diseases

## Abstract

Human endogenous retroviruses (HERVs) comprise approximately 8% of the human genome. Recent studies have considered HERVs as potential pathogenic factors. The majority of HERV genes are mutated and not capable of encoding functional proteins; regardless, some HERV genes, such as HERV-W envelope (env) glycoprotein, are known to have intact open reading frames. The HERV-W element on 7q21.2, which encodes a protein referred to as Syncytin-1, participates in human placental morphogenesis and can activate a pro-inflammatory and autoimmune cascade. Neuropsychological disorders are typically linked to inflammatory abnormalities. In this study, we review that Syncytin-1 has been increasingly involved in the development of neuropsychological disorders, such as schizophrenia and multiple sclerosis (MS). This study also presents inflammation imbalances in schizophrenia and MS. More importantly, we discuss the potential role and molecular mechanisms by which Syncytin-1 regulates inflammatory abnormalities in neuropsychological diseases. In summary, Syncytin-1 activity may represent a novel molecular pathogenic mechanism in neuropyschological diseases, such as schizophrenia and MS.

## Introduction

**Figure 1:**
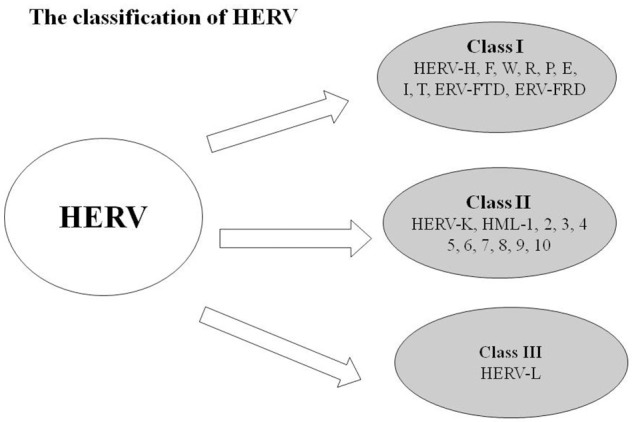
Classifications of HERV: Class I (HERV-H, F, W, R, P, E, I, T, ERV-FTD, and ERV-FRD), Class II (HERV-K, HML-1, 2, 3, 4, 5, 6, 7, 8, 9, 10), and Class III (HERV-L).

Human endogenous retroviruses (HERVs), a class of retroelements, are regarded as remnants of ancient exogenous retroviruses, which integrated into the genome by infecting germ line cells millions of years ago (1). HERVs comprise approximately 8% of the human genome and replicate, along with the human genome, following Mendel's law (2–5). In addition, HERVs are polynucleotide sequences with the complete structure of a retrovirus (6). Classical HERVs have the general components of retroviruses, including the 5′LTR, GAG, POL (retroviral polymerase gene), ENV (envelop), and 3′LTR (7, 8). By phylogenetic analyses of the pol and env genes, HERVs have been identified at least 55 families/groups and categorized into three main classes: Class I (HERV-W and HERV-H), Class II (HERV-K), and Class III (HERV-L; Figure 1). HERV DNA, once classified as useless junk DNA, is essential to human embryonic development and is deeply involved in human evolution.

**Figure 2:**
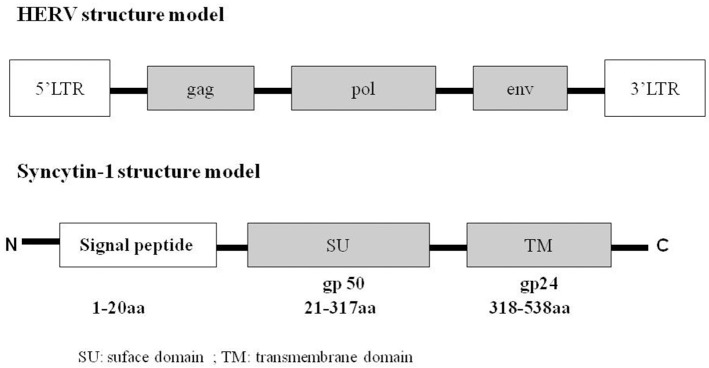
Structure of HERV and Syncytin-1. HERV contains 5′LTR, gag, pol, env, 3′LTR. The env gene, also named Syncytin-1, included signal peptide (1–20 amino acid), SU/gp50 (21–317 amino acid), TM/gp24 (318–538 amino acid). “gp” means glycoprotein.

HERVs include many families, and each has multiple copies. HERV-W, an important member of the HERV family, was first named as multiple sclerosis-associated retrovirus (MSRV). It was isolated from the leptomeningeal choroid plexus, as well as from the Epstein–Barr virus-immortalized B cells of patients with MS (9–12). A complete full-length DNA copy of the HERV-W gene is located at chromosome 7q21, which is defective and does not produce a functional virus (13). Syncytin-1, also known as ERVWE1 or HERV-W Env, is a functional envelope glycoprotein encoded by a single HERV-W env locus that harbors a complete open reading frame (14). Syncytin-1 comprises two functional domains: the cell surface domain (SU) and the transmembrane domain (TM; Figure 2). SU binds with the host cell receptors, and TM promotes virus–cell or cell–cell fusion. Syncytin-1 plays a critical role in placental trophoblastic formation and is involved in the maternal immunosuppressive effect on the fetus. In addition, Syncytin-1 is a highly membranous fusogenic glycoprotein that can induce syncytium formation in cell–cell fusion assay (15, 16). However, recent studies show that Syncytin-1 expression is reproducibly associated with numerous neurological diseases such as schizophrenia, and an increasing number of studies have focused on the potential inflammatory mechanism by which Syncytin-1 mediates neuroimmune activation and oligodendrocyte damage in these diseases. In this article, we mainly introduce the role of Syncytin-1 in inflammatory abnormalities and emphasize an inflammatory mechanism mediated by Syncytin-1 in neuropsychological diseases.

## SYNCYTIN-1 and neurological diseases

Except for the normal physiologic function of Syncytin-1 in the development of placenta, the activity and expression of Syncytin-1 increase in several diseases, such as neuropsychiatric disorders, autoimmune diseases, and cancer (8). Considerably more studies suggest that Syncytin-1 contributes to the development of neuropsychological diseases, such as schizophrenia and MS (3, 17).

### Syncytin-1 and schizophrenia

Schizophrenia is a severe neuropsychiatric disorder characterized by an abnormal social behavior and incapacity to distinguish what is real (18). Findings indicate that a number of genes is contributed to the development of schizophrenia, such as the brain-derived neurotrophic factor (BDNF), neurotrophic tyrosine kinase type 2 receptor (NTRK2), dopamine receptor D3 (DRD3), small conductance Ca^2+^-activated K^+^ channel protein 3 (SK3), and glycogen synthase kinase 3β (GSK3β) (18, 19). Considerable attention has recently been directed toward the role of Syncytin-1 in schizophrenia.

A growing volume of articles have reported the implications of Syncytin-1 in schizophrenia. Syncytin-1 expression in the serum sample of patients with schizophrenia was been described by Perron et al. (20). In the study, positive Syncytin-1 expression was detected in 23 of 49 subjects with schizophrenia but only in 1 of 30 healthy controls. In another research, the transcripts of Syncytin-1 in peripheral blood mononuclear cells (PBMCs) were similarly elevated in patients with schizophrenia relative to those in control subjects (21). In our previous study, we identified the positive mRNA transcription of Syncytin-1 in the plasma samples of 42 in 118 patients with recent-onset schizophrenia; however, none from 106 controls was found (3). We also detected increased protein level of Syncytin-1 in the sera of 99 patients with schizophrenia relative to that of 83 normal individuals by ELISA assay (22). These results, when combined, suggest that Syncytin-1 is involved in the development of schizophrenia.

Several findings contradict the results described above. Frank analyzed Syncytin-1 mRNA expression in the brain of seven healthy individuals and seven individuals with schizophrenia, no differences were found between the groups (23). Meanwhile, similar levels of Syncytin-1 expression were observed in the PBMCs of patients with schizophrenia and controls (24, 25). The inconsistencies with previous findings may be attributable to the following: First, variation in sample size might have influenced the statistical conclusion. Second, the Syncytin-1 expression levels were detected in different tissues and fluids in these studies, such as brain tissue, serum, and PBMCs. The variation in the results for these samples suggested the diverse roles of Syncytin-1 in the development of schizophrenia. Last, the patients with schizophrenia in the studies were at different stages of the disease. In the early and late stages of schizophrenia, pathogenic factors are involved in the promotion and exacerbation of schizophrenia. Therefore, Syncytin-1 can potentially perform different functions in the development of schizophrenia.

### Syncytin-1 and MS

Multiple sclerosis (MS) is a demyelinating disease with chronic inflammation. Patients with MS generally harbor the damaged insulating covers of nerve cells in the brain and spinal cord, disrupting the communication of parts of the nervous system. Consequently, signs and symptoms manifest, including mental, physical, and psychiatric disorders (26). The pathogenesis of MS remains unclear; however, its underlying mechanism involves the destruction of the immune system and the deficiency of myelin-producing cells (27). Potential causes have been identified, which include complex interactions between genetic susceptibility and environmental factors. Viral infection is considered a potential environmental factor (28).

A growing number of studies indicate that Syncytin-1 plays an important role in MS. In 2004, Antony et al. reported that Syncytin-1 was elevated in glial cells in patients with acute demyelination MS. In the aforementioned study, the Syncytin-1 gene was inserted into a virus that could infect astrocytes, and the modified virus was injected into the brains of healthy mice. Overexpression of Syncytin-1 in astrocytes promoted the release of redox reactants cytotoxic to oligodendrocytes. Two weeks post-injection, the mice developed MS-like symptoms, and numerous deformed and dead oligodendrocytes were found during autopsy (29). Perron et al. also found the physiologic expression of HERV-W in gray matter and white matter microglia as well as in central vascular endothelial cells in patients with MS (30). In 2007, Giuseppe et al. demonstrated that HERV-W env (Syncytin-1) and pol genes were highly expressed in the brain and PMBCs of individuals with MS by polymerase chain reaction and reverse transcription–PCR. Immunohistochemical analysis showed that the protein level of Syncytin-1 was only expressed in the glial cells of patients with MS exhibiting hyperplastic damage and was mainly distributed in the margins of microglia and astrocytes (31). In 2010, MSRV was observed in the cerebrospinal fluid of patients with early MS and contributed to the secondary progressive phase of MS (32). Given the role of Syncytin-1 in MS has been widely acknowledged; thus, the study of Syncytin-1 may provide new ideas for defining the neuropathic mechanisms of MS as well as its diagnosis, prognosis, and treatment (33, 34).

## Inflammatory abnormalities in neuropsychological diseases

Inflammation is a series of complex biological reactions of an organism in response to harmful stimuli (35). Various inflammatory cytokines, which play a role in initiating the inflammatory response, are essential for regulating inflammation (36). In the central nervous system (CNS), the inflammatory cytokines produced by neuronal and glial cells affect the brain cortical neuronal development (37–39). Inflammatory abnormalities are involved in a wide range of human diseases and are regarded as the potential pathogenesis of neuropsychological diseases such as schizophrenia (40) and MS (41).

### Inflammation and schizophrenia

Inflammatory abnormalities have been repeatedly linked to schizophrenia in recent research (42–44). In either the early pivotal stage of brain development or the adult acute disease state, inflammation significantly affects the development of schizophrenia (36).

A confluence of evidence has demonstrated an association between prenatal inflammation induced by bacterial or viral agent infections and increased risk of schizophrenia in the offspring during adulthood (36, 45, 46). Studies on rodents have indicated that an immune disorder during pregnancy can result in mimic clinical symptoms of schizophrenia in the adult offspring, including brain dysfunction and behavioral changes (47). The correlation between inflammation and schizophrenia developed in adulthood has been investigated, in addition to that in prenatal and perinatal inflammation (48–51). Inflammatory response in the development of schizophrenia is a chronic low-grade response rather than an acute and short-term status (51, 52). Acute inflammation is a quick response and often benefits tissue repair and recovery (53, 54), whereas chronic inflammation has long-term consequences that are often detrimental, inducing immune system perturbations (55, 56). This finding may be one of the reasons that high rates of chronic inflammation are reported in patients with schizophrenia. Numerous studies have revealed increased concentrations of several inflammatory cytokines in the patients with schizophrenia (57). Increased levels of nterleukin (IL) 1-β, IL-2, IL-6, IL-8, IL-12, transforming growth factor-beta (TGF-β), and tumor necrosis factor-alpha (TNF-α) were detected in patients with schizophrenia than in controls (52, 58–60). C-reactive protein (CRP), another pro-inflammatory molecule, has recently been found to be sufficiently increased in patients with schizophrenia (36, 61–63). An underlying mechanism of inflammatory cytokine contributing to schizophrenia is apoptosis, which can induce neuronal injury or death (64, 65). Researchers have demonstrated that the alteration in the apoptotic cascade can potentially lessen the viability of neuron and glia at different stages of neurodevelopment, inducing the deficits in brain volume and function in schizophrenia (66–68). Another mechanism is that chronic inflammation may induce the damage of the brain microvascular system and disruption of the blood–brain barrier and cerebral blood flow, which may lead to the development of clinical symptoms of schizophrenia (69–71). Thus, inflammation plays a critical role in the development of schizophrenia.

### Inflammation and MS

MS is a chronic inflammatory demyelinating disorder. Inflammatory disorders play a pivotal role in MS. Richard et al. found significantly increased secretions of inflammatory cytokines IL-1β and TNF-α in the monocytes of patients with MS relative to those of the controls (72). Meanwhile, **Celia** et al. performed immunohistochemistry to detect the expression and distribution of pro-inflammatory and regulatory cytokines in different MS lesions and compared the inflammatory or non-inflammatory components of CNS tissues with other neurological diseases. Results showed a widespread distribution of cytokines in perivascular inflammatory cells and glial cells in all inflammatory lesions. No apparent pattern of these cytokines in MS lesions were observed; however, pro-inflammatory cytokines were rarely detectable under normal and non-inflammatory conditions, and regulatory cytokines were easily detected in MS (73). Moreover, Josa et al. observed the robust brain inflammation response in the relapsing–remitting MS (RRMS), secondary progressive MS (SPMS), and primary progressive MS(PPMS). An evidently significant correlation between inflammation and axonal injury was observed in both the global MS population and progressive MS alone (74). These results indicate that inflammation is associated with MS and depict a potential process of inflammation triggered in MS. During the inflammatory reaction, encephalitogenic lymphocytes, which are activated peripherally, bind to receptors of endothelial cells within the CNS and then cross the blood–brain barrier, pass into the interstitial matrix, and trigger and amplify the inflammatory disorders in the brain. Inflammatory abnormalities may further induce neurodegeneration in MS (75).

## SYNCYTIN-1 could cause inflammatory abnormalities in neuropsychological diseases

Recent research has linked HERVs to the inflammatory condition in neuropsychological diseases. HERV-K, another most studied HERV, was found to have a robust expression in the brain of subjects with amyotrophic lateral sclerosis (ALS) (76, 77). In addition, the inflammatory transcription factors interferon regulatory factor 1 (IRF1) and NF-κB could trigger the HERV-K expression via its interferon-stimulated response elements in neurons of the motor cortex in ALS (78), suggesting the potential role of HERVs in mediating inflammation in neuropsychological diseases.

Syncytin-1, functioning as an immunotoxin, can induce inflammation with superantigen-like effects, thereby activating the innate immune system (79). Studies indicate that specific infections can activate HERV-W elements, leading to the production of Syncytin-1, which then stimulates pro-inflammatory and neurotoxic cascades (21). Murphy demonstrated that overexpression of Syncytin-1 upregulated the expression of proinflammatory factors, such as IL-1β and IL-6 (80). Moreover, Syncytin-1 overexpression in glial cells can trigger endoplasmic reticulum stress, leading to neuroinflammation and the production of free radicals to destroy proximate cells (34). Given the regulatory role of Syncytin-1 in inflammation, abnormal expression of Syncytin-1 may result in cell death or tissue damage (81). An *in vitro* study indicates indirect cytotoxicity of Syncytin-1 to oligodendrocytes, and murine models show that Syncytin-1 overexpression can lead to demyelination (17, 29, 31, 82). In a study by Perron, Syncytin-1 not only induced proinflammatory reaction but also exhibited the ability to trigger experimental autoimmune encephalomyelitis (EAE) in mice (83). Owing to its potential to elicit immunosuppressive and neuroinflammatory effects, Syncytin-1 has been linked to some neurological and neuropsychiatric disorders (29, 84). For instance, Syncytin-1 has been regarded as an important regulator in the development of MS and schizophrenia because of its capacity to induce neuroinflammation and cytotoxicity. In the present study, we introduce several potential mechanisms of Syncytin-1 involved in neuroinflammation.

### Syncytin-1 increases nitric oxide in glial cells

Schizophrenia and MS are neurological diseases with an inflammatory response in the the CNS (85). Glial cells, including astrocytes, microglia, and oligodendroglial cell, are widespread in the CNS and are necessary for regulating brain inflammation (86). Nitric Oxide (NO) plays regulatory roles in the inflammatory condition of the brain and the function of neuronal cells and participates in the pathogenesis of various neuropsychological diseases (87, 88). Antony et al. indicated that Syncytin-1 could activate the inducible NO synthase in astrocytes to initiate an old astrocyte specifically induced substance (OASIS)-mediated suppression of ASCT1 (17). In addition, Antony et al. observed that the overexpression of Syncytin-1 in astrocytes also induced the release of the oxidation–reduction reaction product and NO, which exhibited cytotoxicity to oligodendrocytes (29). In our recent research, we found that overexpression of Syncytin-1 in microglia could induce the expression of inducible NO synthase to increase NO production and promote the migration of microglia (89). This combination allows Syncytin-1 to contribute to neuroinflammation by inducing the production of NO in glial cells.

### Syncytin-1 induces proinflammatory cytokines via CD14 and TLR4 in human monocytes

Toll-like receptor 4 (TLR4) is a transmembrane protein belonging to the TLR family. It can recognize lipopolysaccharide and lead to the activation of the NF-κB signal transduction pathway and the production of inflammatory cytokines. TLR4 mainly participates in activating the innate immune system. Meanwhile, CD14 is a glycosylphosphatidylinositol-anchored membrane protein, which functions as a pattern recognition receptor with the extracellular domain of TLR4. A referenced article focused on the inflammatory response induced by Syncytin-1 and CD14-TLR4. In human monocytes, activation of Syncytin-1 could induce the proinflammatory cytokines IL-6, IL-1β, and TNF-α; however, the incubation of the neutralizing antibodies of CD14 and TLR4 effectively blocked the secretion of these cytokines (90). The signaling pathways of CD14 and TLR4 in glial cells have not been confirmed; regardless, increased TLR4 has been identified in the oligodendroglial cell of MS, inducing brain inflammation (29). Moreover, the proinflammatory cytokines IL6, IL-1β, and TNF-α are important for regulating the inflammation status in the CNS and brain development (39, 91, 92). Given these findings, we consider that CD14/TLR4 potentially mediates Syncytin-1 in the CNS to induce proinflammatory cytokines and participates in neuropsychological diseases, such as schizophrenia and MS.

### Syncytin-1 induces CRP activation via TLR3 in glial cells

C-reaction protein (CRP), an inflammatory marker, is associated with several neuropsychological diseases. For instance, CRP was elevated in the serum of patients with schizophrenia (93) and MS (94). Recent research indicated that the expression of several TLRs, including TLR3, was highly increased in the blood of individuals with schizophrenia (95). A member of the TLR family, TLR3 mainly recognizes the virus dsDNA and activates the innate immune system. Activation of TLR3 can induce the production of proinflammatory cytokines as diverse as IL-6, IL-1β, and TNF-α (96, 97). In our recent study, we reported that Syncytin-1 exhibited a positive correlation and marked consistency with the expression levels of CRP in individuals with schizophrenia. We also found that Syncytin-1 could trigger the activation of CRP via the TLR3-IL-6 signal pathway in glial cells, the deficiency of TLR3 could significantly impair Syncytin-1-induced CRP and IL-6 expression (22). Direct interaction and cellular colocalization between Syncytin-1 and TLR3 were observed by confocal microscopy (22). Thus, TLR3 can potentially function as a Syncytin-1 mediator to induce inflammatory abnormalities in the glial cell.

### HLA-A^*^0201^+^-restricted epitopes of syncytin-1 could induce cytotoxic T lymphocytes

HLA-A^*^0201^+^ is a human leukemia antigen. HLA restriction is involved in the immune response to neuropsychiatric diseases. The epitopes derived from Syncytin-1 were the HLA-A^*^0201 restriction and potential for adoptive immunotherapy. In the study, we predicted and synthesized five peptides that displayed HLA-A^*^0201-binding motifs of Syncytin-1. Among the peptides, peptides W, Q and T could promote the proliferation of lymphocytes. The stimulation of these peptides on PBMSs from HLA-A^*^0201^+^ donors could induce peptide-specific CD8^+^ T cells. Abundant interferon-γ-secreting T cells were also detected after stimulation of these peptides for several weeks. These data demonstrate that Syncytin-1 peptides (such as W, Q, and T peptides) can induce HLA-A2.1-restricted CD8^+^ CTL and could be a potential target for astrocytoma immunotherapy (98). On the other hand, the cytotoxic T lymphocytes induced by Syncytin-1 could be a potential mechanism for inflammatory abnormalities in the CNS.

## Comments

Recent clinical reports have indicated the importance of Syncytin-1 in neuropsychological diseases; regardless, these studies have several limitations. First, the sample sizes of these clinical studies are relatively small. The sample size is crucial because an insufficient sample size may render testing and reproduction for statistical significance difficult. An increasing sample size is necessary for verifying the role of Syncytin-1 in these diseases. Second, other psychiatric control groups in a clinical study can benefit from the enhancement of the potential implications of the findings.

A notable finding from the previous observations is that the abnormal expression levels of Syncytin-1 in neuropsychological diseases seem ubiquitous. For instance, elevated Syncytin-1 expression in MS was detected in different tissues or fluids, including the serum, PBMCs, glial cells, and brain tissues from patients with MS (31, 32, 99, 100). This elevation presents a challenge for clearly elaborating on the pathogenic mechanism of Syncytin-1 in neuropsychological diseases. It also suggests that Syncytin-1 can execute multiple functions in the development of diseases. The evidence in relation to the association between Syncytin-1 and inflammation demonstrates that the change in Syncytin-1 in neuropsychological diseases seems not to be an incidental phenomenon. In view of the brain damage in neuropsychological diseases, increased Syncytin-1 in the cerebrospinal fluid or neurogliacyte may be associated with neuroinflammation, leading to brain injury. Our previous data supported this possibility. Our study found that Syncytin-1 could trigger the production of inflammatory cytokines CRP and IL-6 in microglial and astroglial cells (22). Another study also suggested that Syncytin-1 can induce inflammation by promoting the secretion of IL-6, IL-1β, and TNF-α in human monocytes (90). Owing to the association between Syncytin-1 and inflammation, abnormal **S**yncytin-1 expression in PBMCs indicates that Syncytin-1 may promote the inflammatory stage of immune cells in blood, enhancing inflammation in the brain of individuals with neuropsychological diseases. Therefore, the role of Syncytin-1 in neuropsychological diseases may be complex, and more clinical studies and cells experiments are necessary to verify the specific functions of Syncytin-1 in different tissues or fluids in neuropsychological diseases.

In the molecular mechanisms of Syncytin-1 regulating inflammation in neuropsychological diseases, TLRs may be the essential factors. Once activated, TLR3 and TLR4 can trigger the innate immune reaction. We found that TLR3 could mediate the inflammatory effect of Syncytin-1 in microglial and astroglial cells (22). Meanwhile, the neutralizing antibodies of TLR4 could effectively impair the inflammation induced by Syncytin-1 in human monocytes (90). Therefore, different TLRs may function as mediators to induce inflammation reaction in response to Syncytin-1 in different tissues or fluids. Regardless, the existing data in the literature remain inconclusive. The mechanism of Syncytin-1 regulation of neuroinflammation in neuropsychological diseases has yet to be elucidated. Further research on the role of Syncytin-1 in neuropsychological diseases has to be conducted. Mouse models should also be used in these studies.

## Conclusion

**Figure 3:**
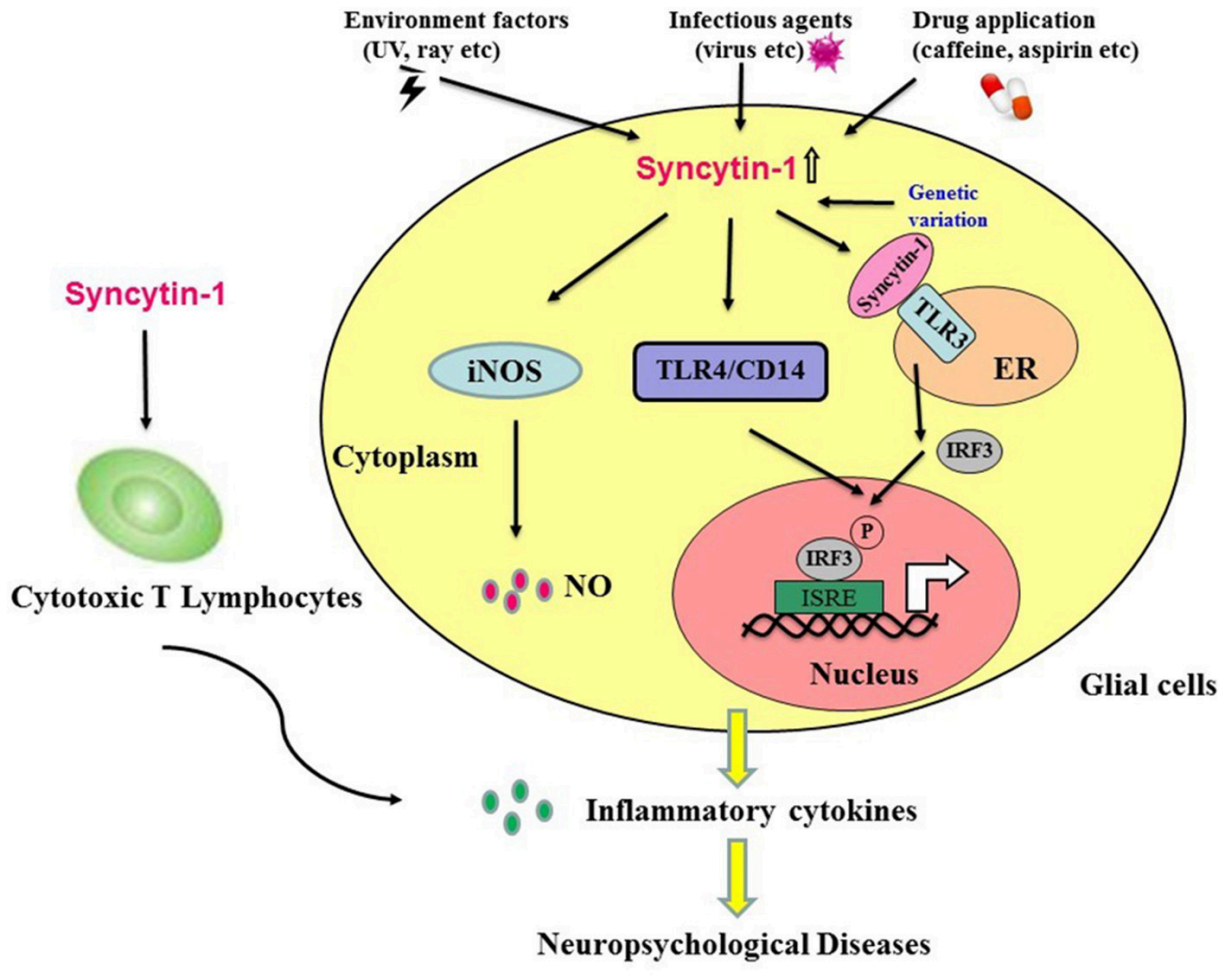
Hypothesis that Syncytin-1 contributes to inflammatory abnormalities, which lead to neuropsychological diseases. Environmental factors (e.g., ultraviolet rays), infectious agents (e.g., viruses), drug application (e.g., caffeine, aspirin, etc.), and genetic variation could trigger the expression of Syncytin-1 in glial cells. The expression of Syncytin-1 induced the release of nitric oxide in microglia and astrocytes and activated the TLR signaling pathways (e.g., TLR3 and TLR4) to induce the production of inflammatory cytokines. In addition, Syncytin-1-derived cytotoxic T lymphocytes could also secrete inflammatory cytokines. The production of these inflammatory cytokines led to the inflammatory abnormalities in the CNS and contributed to the development of neuropsychological diseases.

An increasing number of findings suggest that neuropsychological diseases result from both genetic and environmental factors. In addition to genetic factors, environmental factors play an essential role in disease development, particularly in the early phases of brain neurodevelopment (18, 19). Syncytin-1 may link environmental and genetic factors. Accumulating evidence indicates that Syncytin-1 is closely involved in the development of neuropsychological diseases. Environmental factors, such as specific viral infections, drug application, and exposure to ultraviolet rays (22), can induce Syncytin-1. The elevated Syncytin-1 in the brain has been associated with abnormal inflammation, contributing to the development of neuropsychological diseases. Many studies reveal the potential role of Syncytin-1 in neuroinflammation, but the potential mechanisms of HERV pathogenicity have yet to be elucidated. In this study, we described several activated signaling networks in response to Syncytin-1 that may lead to abnormal inflammation in neuropsychological diseases: Syncytin-1 may induce the inflammatory abnormalities via four routes: (1) release of NO; (2) activation of the TLR4/CD4 pathway; (3) activation of the TLR3 signal pathway; (4) induction of CTL. These inflammatory abnormalities could lead to neuronal damage and apoptosis of neuron cells, which play crucial roles in neuropsychological diseases such as schizophrenia and MS (Figure 3).

We summarize the relationship between increased Syncytin-1 and abnormal inflammation and elucidate the potential mechanisms of inflammation induced by Syncytin-1 in neuropsychological disorders. This review also presents a new insight into the diagnosis and treatment of neuropsychological diseases.

## Author contributions

XW prepared the first draft of the manuscript, which was corrected and improved by JH and FZ. All authors read and approved the final manuscript.

### Conflict of interest statement

The authors declare that the research was conducted in the absence of any commercial or financial relationships that could be construed as a potential conflict of interest.
